# Supplementary material for: Comparative multiomics analysis of cell physiological state after culture in a basket bioreactor
Source: Sci Rep. 2022 Nov 23;12:20161. doi: 10.1038/s41598-022-24687-4 (PMC9686226; doi:10.1038/s41598-022-24687-4)
Supplement: Supplementary file 1 — Supplementary Information 1. [file 41598_2022_24687_MOESM1_ESM.zip › raw data/Transcriptomic raw data/6.Enrichment/1.GO/TE1vsTC1/GSEA/TC1/NUCLEOTIDE_CATABOLIC_PROCESS/NUCLEOTIDE_CATABOLIC_PROCESS(GO_0009166).html]

Details for gene set NUCLEOTIDE\_CATABOLIC\_PROCESS(GO:0009166)[GSEA]


|  || Dataset | readcount.sample |
| Phenotype | sample.cls |
| Upregulated in class | TC1 |
| GeneSet | NUCLEOTIDE\_CATABOLIC\_PROCESS(GO:0009166) |
| Enrichment Score (ES) | -0.1537255 |
| Normalized Enrichment Score (NES) | -0.8396472 |
| Nominal p-value | 0.52134144 |
| FDR q-value | 0.97238207 |
| FWER p-Value | 1.0 |
Table: GSEA Results Summary

  

Fig 1: Enrichment plot: NUCLEOTIDE\_CATABOLIC\_PROCESS(GO:0009166)
  
Profile of the Running ES Score & Positions of GeneSet Members on the Rank Ordered List

  

| PROBE | DESCRIPTION   (from dataset) | GENE SYMBOL | GENE\_TITLE | RANK IN GENE LIST | RANK METRIC SCORE | RUNNING ES | CORE ENRICHMENT || 1 | 103232107 | LOC103232107 |  |  | 235 | Infinity | -0.0100 | Yes |
| 2 | 119623417 | LOC119623417 |  |  | 1788 | 2.248 | 0.0353 | Yes |
| 3 | 103217648 | TMEM237 |  |  | 4110 | 1.514 | 0.0087 | Yes |
| 4 | 103242214 | ENO3 |  |  | 4874 | 1.414 | 0.0467 | Yes |
| 5 | 103238217 | PFKM |  |  | 6902 | 1.229 | 0.0188 | Yes |
| 6 | 103240047 | LOC103240047 |  |  | 7696 | 1.166 | 0.0428 | Yes |
| 7 | 103230811 | EDARADD |  |  | 12094 | 0.889 | -0.1084 | Yes |
| 8 | 103232238 | PGK1 |  |  | 12477 | 0.864 | -0.0814 | Yes |
| 9 | 103236549 | LOC103236549 |  |  | 12544 | 0.860 | -0.0406 | Yes |
| 10 | 103243434 | LOC103243434 |  |  | 12790 | 0.845 | -0.0084 | Yes |
| 11 | 103237780 | LOC103237780 |  |  | 13638 | 0.793 | -0.0059 | Yes |
| 12 | 103230584 | LOC103230584 |  |  | 14738 | 0.725 | -0.0180 | Yes |
| 13 | 119623700 | LOC119623700 |  |  | 14837 | 0.718 | 0.0142 | Yes |
| 14 | 103216571 | ENO4 |  |  | 14869 | 0.716 | 0.0493 | Yes |
| 15 | 103217002 | LOC103217002 |  |  | 15680 | 0.663 | 0.0469 | Yes |
| 16 | 103236321 | LOC103236321 |  |  | 15792 | 0.656 | 0.0754 | Yes |
| 17 | 103246072 | PKM |  |  | 16089 | 0.635 | 0.0945 | Yes |
| 18 | 103219799 | PFKL |  |  | 16212 | 0.628 | 0.1211 | Yes |
| 19 | 103234470 | GPI |  |  | 16493 | 0.605 | 0.1394 | Yes |
| 20 | 103242471 | NT5M |  |  | 17742 | 0.511 | 0.1097 | No |
| 21 | 119621354 | LOC119621354 |  |  | 18273 | 0.463 | 0.1096 | No |
| 22 | 119618100 | ALDOA |  |  | 18765 | 0.414 | 0.1088 | No |
| 23 | 103240043 | NT5E |  |  | 19263 | 0.364 | 0.1052 | No |
| 24 | 103218486 | ENO2 |  |  | 19420 | 0.349 | 0.1160 | No |
| 25 | 103243056 | NT5C |  |  | 20232 | 0.236 | 0.0918 | No |
| 26 | 103242625 | ALDOC |  |  | 20901 | 0.100 | 0.0670 | No |
| 27 | 103219114 | ALDOB |  |  | 22052 | 0.000 | 0.0156 | No |
Table: GSEA details
[plain text format]

  

Fig 2: NUCLEOTIDE\_CATABOLIC\_PROCESS(GO:0009166)
  
Blue-Pink O' Gram in the Space of the Analyzed GeneSet

  

Fig 3: NUCLEOTIDE\_CATABOLIC\_PROCESS(GO:0009166): Random ES distribution
  
Gene set null distribution of ES for
 **NUCLEOTIDE\_CATABOLIC\_PROCESS(GO:0009166)**
